# Supplementary material for: Non-homologous end joining shapes the genomic rearrangement landscape of chromothripsis from mitotic errors
Source: Nat Commun. 2024 Jul 4;15:5611. doi: 10.1038/s41467-024-49985-5 (PMC11224358; doi:10.1038/s41467-024-49985-5)
Supplement: Supplementary file 1 — Supplementary Information [file 41467_2024_49985_MOESM1_ESM.pdf]

Inventory of Supplementary Information for Hu et al.

Supplementary Fig. 1. Generation of DSB repair knockout cells by CRISPR/Cas9 editing.

Supplementary Fig. 2. Induction of micronuclei and chromosome fragmentation in knockout cells.

Supplementary Fig. 3. Genomic rearrangement landscape of mis-segregated chromosomes following transient centromere inactivation.

Supplementary Fig. 4. Genomic rearrangement landscape of mis-segregated chromosomes following sustained centromere inactivation.

Supplementary Fig. 5. Characterization of genomic rearrangements in DNA-PKcs-deficient cells.

Supplementary Fig. 6. Complex rearrangements in DNA-PKcs-deficient cells arise from functional redundancies of the NHEJ pathway.

Supplementary Fig. 7. Nuclear envelope rupture triggers the loss of DNA-PKcs from micronuclei.

Supplementary Fig. 8. Depletion of DNA-PKcs prolongs 53BP1 residence time at MN bodies and triggers cell cycle arrest.

Supplementary Fig. 9. Characterization of calyculin A-induced premature chromosome condensation.

Supplementary Fig. 10. Induction of genome-wide or Cas9-induced DNA breaks on nuclear chromosomes facilitates translocations involving the micronucleated chromosome.

Supplementary Table 1. List of oligonucleotide sequences used in this study.

Supplementary Table 2. List of primary antibodies used in this study.

Source Data containing scans of uncropped blots and flow cytometry gating strategies

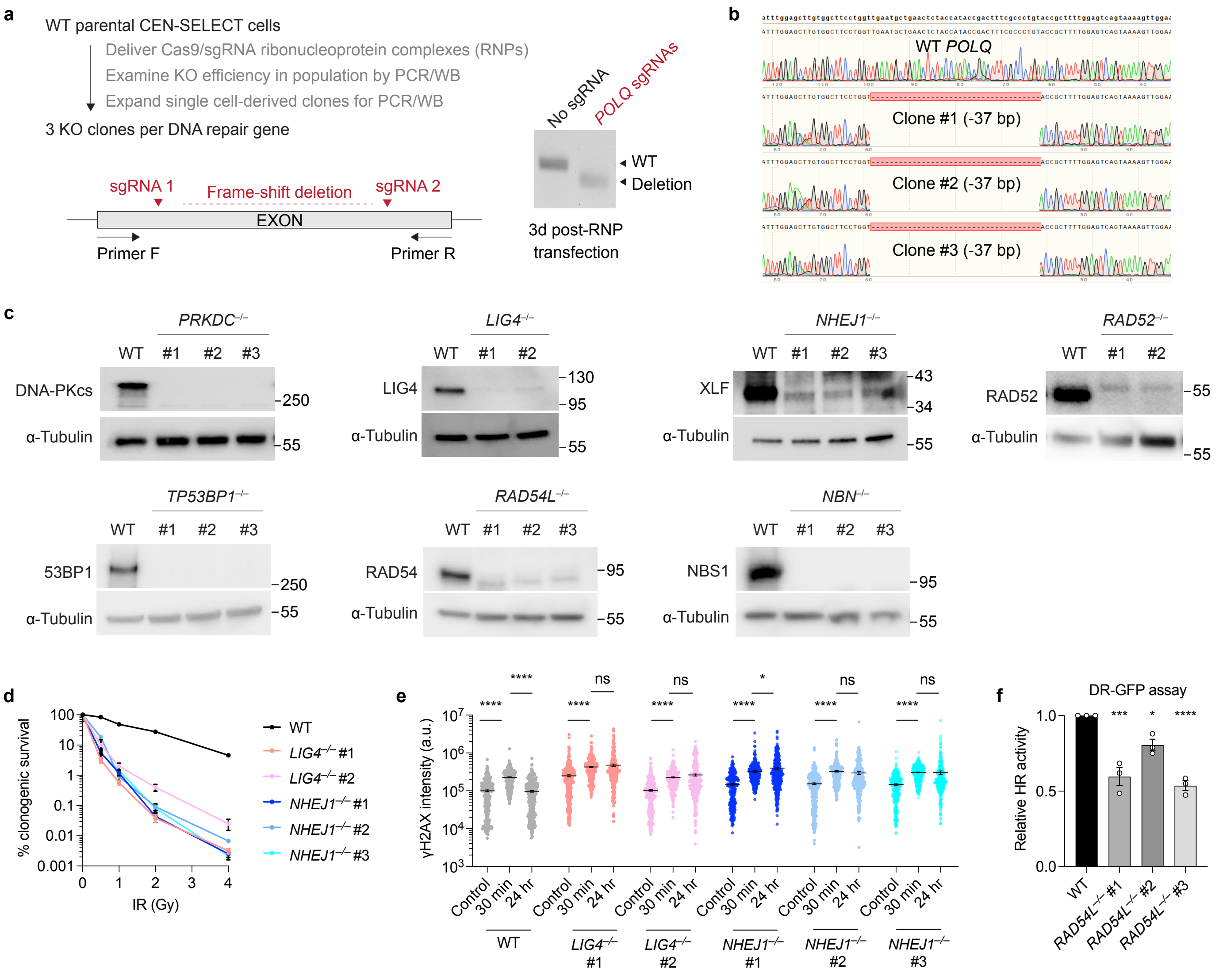

Supplementary Fig. 1. Generation of DSB repair knockout cells by CRISPR/Cas9 editing.
a) Experimental schematic for generating CRISPR/Cas9-mediated biallelic knockout clones. Cleavage at two sgRNA sequences yields a frameshift deletion that can be detected by PCR. **b)** Sanger sequencing confirmation of predicted 37 base pair frameshift deletion in the *POLQ* gene in three independent clones. **c)** Confirmation of KO clones by immunoblotting. Molecular weight markers are indicated in kilodaltons. **d)** Clonogenic assay following exposure of LIG4 and XLF KO clones to the indicated doses of ionizing radiation (IR). **e)** Fluorescence intensity of γH2AX in the nucleus of LIG4 and XLF KO clones following exposure to 1 Gy IR and recovery for 30 min or 24 hrs. Controls represent non-irradiated cells. Data pooled from (left to right): n = 303, 325, 311, 326, 310, 250, 304, 317, 309, 301, 297, 304, 321, 315, 297, 301, 323, and 297 cells. Negative values were excluded from the plot but considered in the statistical analysis. **f)** Measurement of relative levels of homologous recombination in RAD54 KO clones using the DR-GFP assay. Data represent the mean ± SEM from n = 3 independent experiments. Statistical analyses were calculated by ordinary one-way ANOVA test with multiple comparisons. ns, not significant; *$p \leq 0.05$; **$p \leq 0.01$; ***$p \leq 0.001$; ****$p \leq 0.0001$. Source data are provided as a Source Data file.

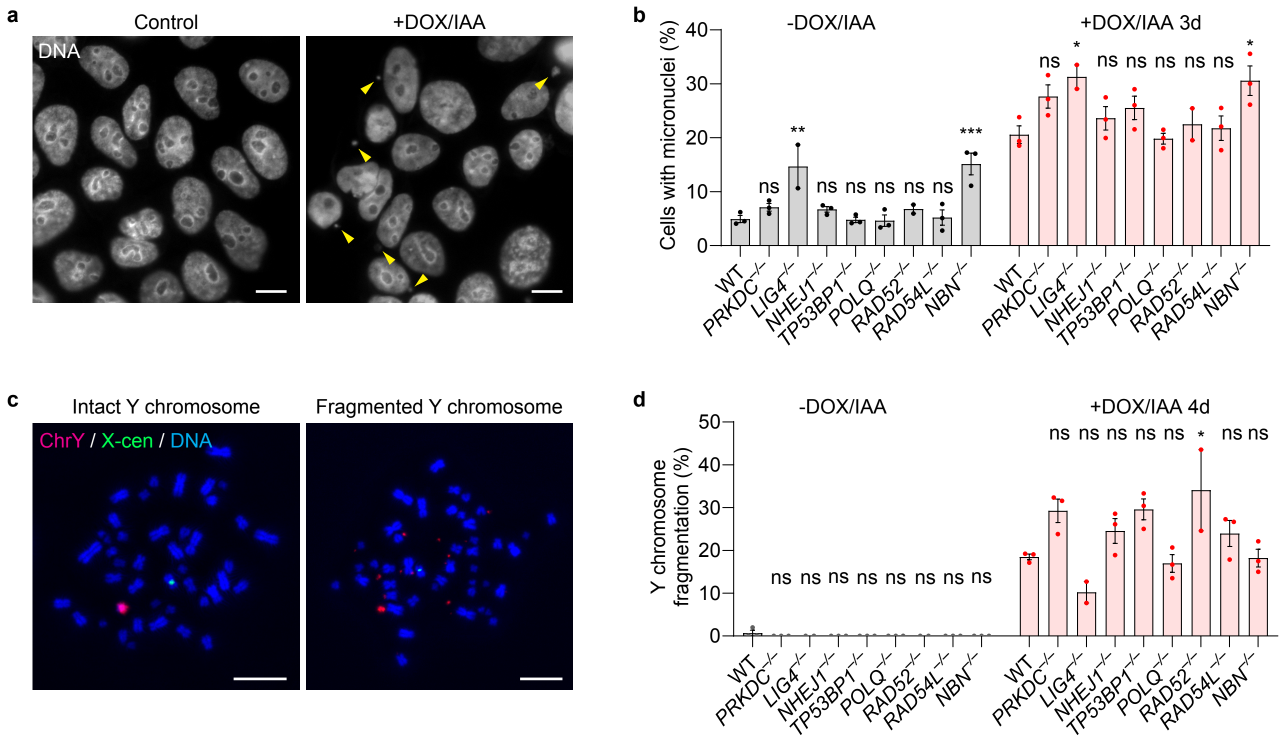

Supplementary Fig. 2. Induction of micronuclei and chromosome fragmentation in knockout cells.
a) Representative images of DNA-PKcs KO cells with micronuclei before and after induction with DOX/IAA. Scale bar, 10 μm. **b)** The percentage of cells with micronuclei with and without 3d DOX/IAA treatment. Data pooled from (left to right): 1,367, 1,711, 2,208, 1,236, 340, 407, 1,554, 1,709, 396, 335, 1,985, 1,769, 935, 295, 1,256, 1,782, 706, and 736 cells. **c)** Images of metaphase spreads with intact or fragmented Y chromosomes after 4d DOX/IAA treatment. Scale bar, 10 μm. **d)** Frequency of Y chromosome fragmentation. Only Y chromosome-positive metaphase spreads were scored. Data pooled from (left to right): 168, 267, 113, 226, 81, 141, 162, 162, 148, 147, 184, 213, 79, 123, 120, 186, 215, and 255 metaphase spreads. Bar graphs in **(b)** and **(d)** represent the mean ± SEM from n = 3 independent experiments for WT controls, n = 2 KO clones for LIG4 and RAD52, and n = 3 KO clones for DNA-PKcs, XLF, 53BP1, Polθ, RAD54, and NBS1; statistical analyses were calculated by ordinary one-way ANOVA test with multiple comparisons. ns, not significant; *$p \leq 0.05$; **$p \leq 0.01$; ***$p \leq 0.001$; ****$p \leq 0.0001$. Source data are provided as a Source Data file.

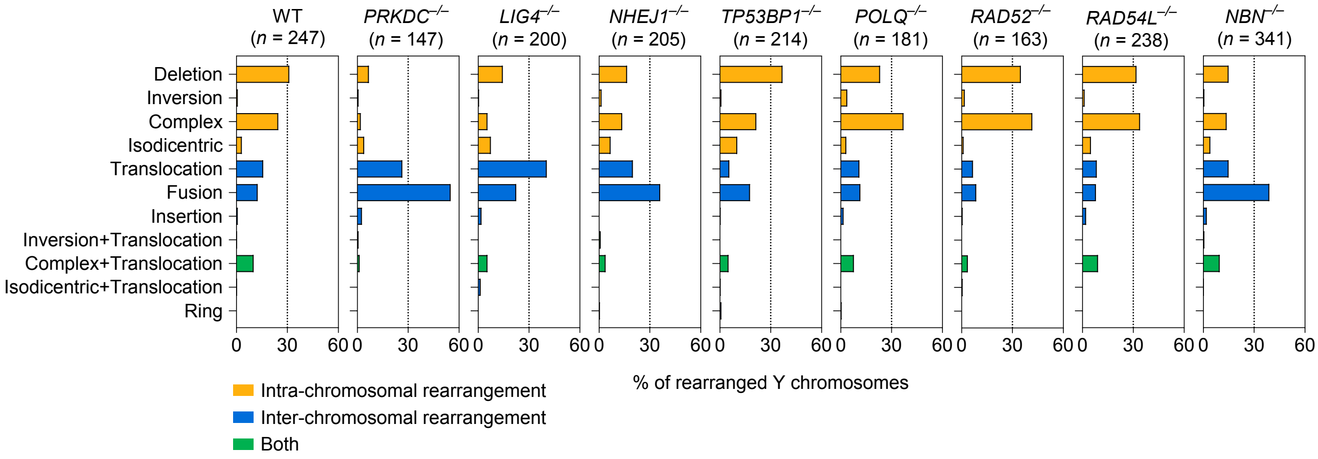

Supplementary Fig. 3. Genomic rearrangement landscape of mis-segregated chromosomes following transient centromere inactivation. Distribution of Y chromosome rearrangement types as determined by metaphase FISH following 3d DOX/IAA treatment and G418 selection. Sample sizes indicate the number of rearranged Y chromosomes examined; data are pooled from 3 independent experiments for WT cells and 2-3 independent KO clones per gene. Source data are provided as a Source Data file.

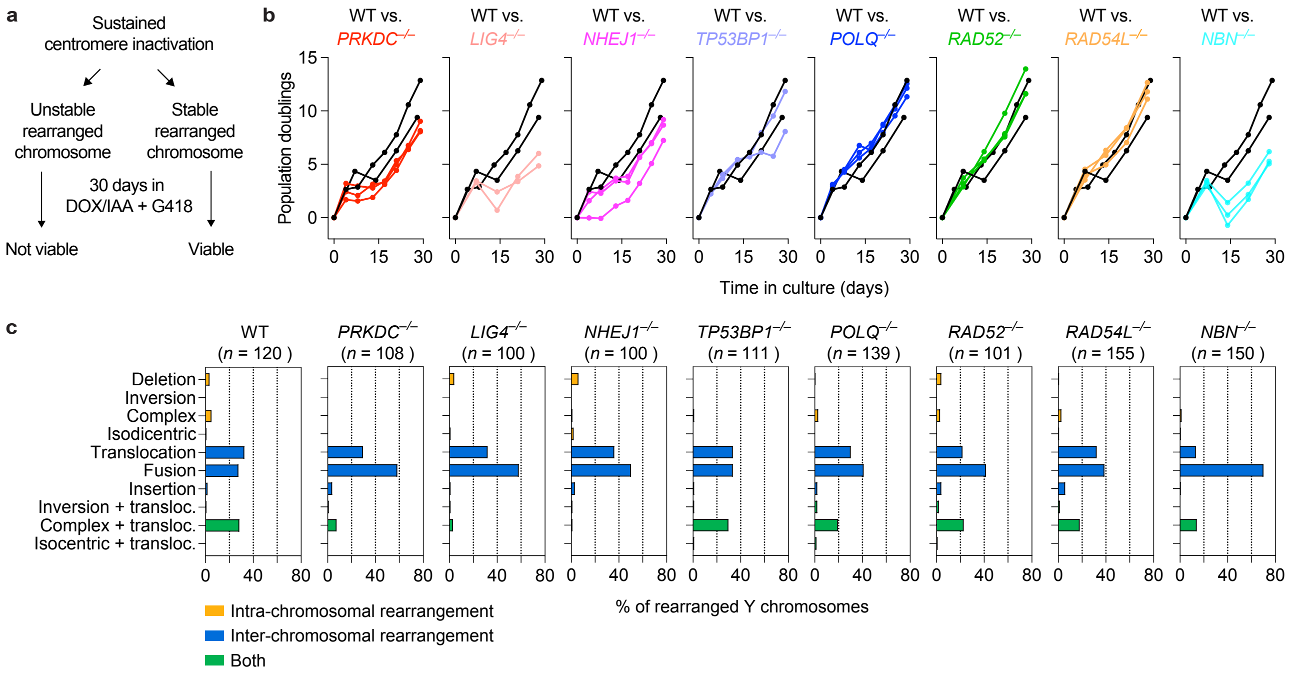

Supplementary Fig. 4. Genomic rearrangement landscape of mis-segregated chromosomes following sustained centromere inactivation.
a) Schematic of turnover between proliferating cells harboring stable Y chromosome rearrangements and dying cells that are unable to maintain the Y-encoded neoR marker. **b)** Plots of cumulative cell doublings over a 30d period in the presence of DOX/IAA and G418. Each curve represents an individual clone except for WT, which represent independent experiments using the same cell line. **c)** Distribution of Y chromosome rearrangement types as determined by metaphase FISH following continuous passaging in DOX/IAA and G418. Sample sizes indicate the number of rearranged Y chromosomes examined; data are pooled from 2 independent experiments for WT cells or 2-3 independent KO clones per gene. Source data are provided as a Source Data file.

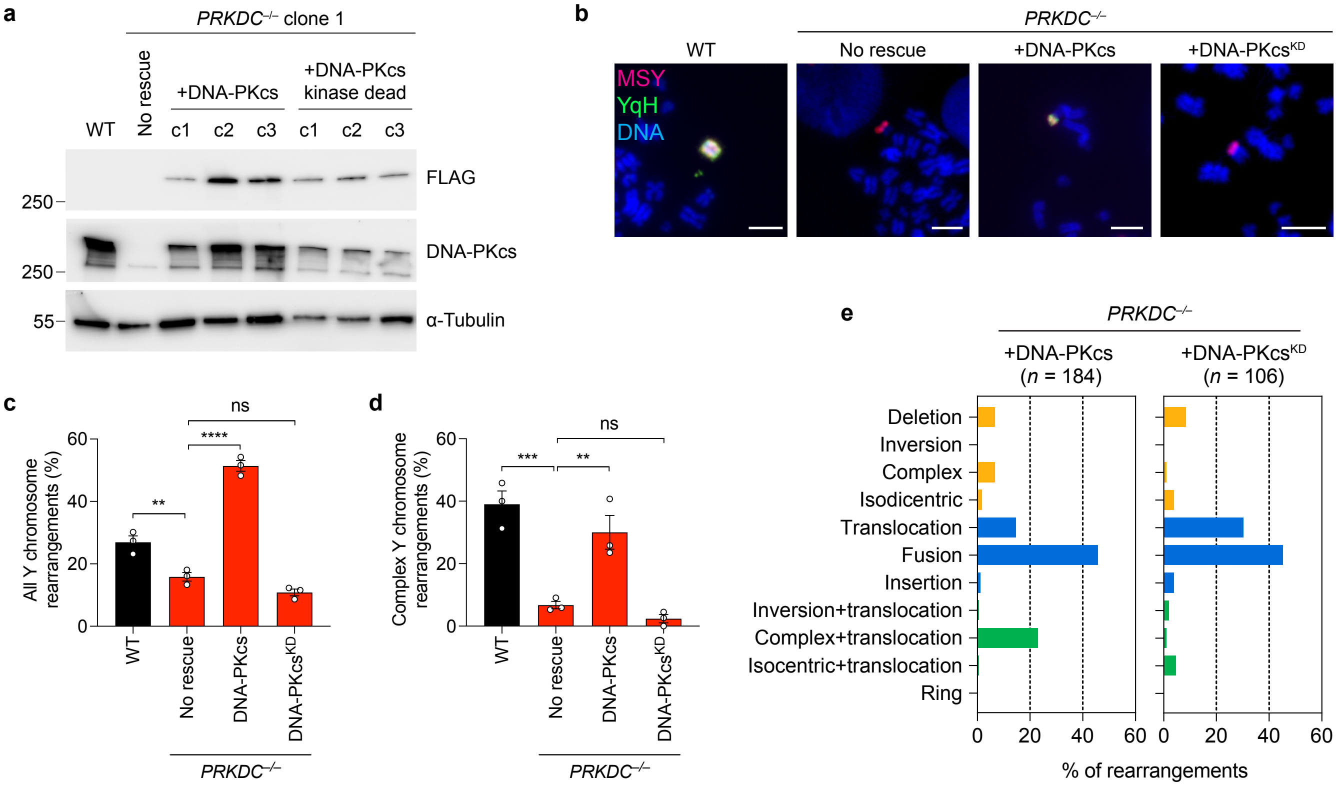

Supplementary Fig. 5. Characterization of genomic rearrangements in DNA-PKcs-deficient cells.
a) Immunoblot confirmation of DNA-PKcs KO cells expressing either FLAG-tagged WT (WT) or kinase-dead (KD) DNA-PKcs. Molecular weight markers are indicated in kilodaltons. **b)** Examples of Y chromosome rearrangements detected by metaphase FISH using the indicated sets of probes. Scale bar, 5 µm. **c)** Frequency of Y chromosome rearrangements in DNA-PKcs KO cells complemented with WT or kinase-dead (KD) DNA-PKcs. Data are pooled from (left to right): 195, 339, 390, and 365 metaphase spreads. **d)** Proportion of Y chromosome rearrangements that can be classified as complex in DNA-PKcs KO cells complemented with WT or KD DNA-PKcs. Data are pooled from (left to right): 70, 80, 184, and 106 metaphases spreads with Y chromosome rearrangements. Bar graphs in panels **(c-d)** represent the mean ± SEM of n = 3 independent experiments for WT and DNA-PKcs KO, n = 3 independent DNA-PKcs rescue clones. Statistical analyses were calculated by ordinary one-way ANOVA test with multiple comparisons. ns, not significant; $^{**}p \leq 0.01$; $^{***}p \leq 0.001$; $^{****}p \leq 0.0001$. **e)** Distribution of Y chromosome rearrangement types as determined by metaphase FISH following 3d DOX/IAA treatment and G418 selection. Sample sizes indicate the number of rearranged Y chromosomes examined; data are pooled from 3 independent clones per condition. Source data are provided as a Source Data file.

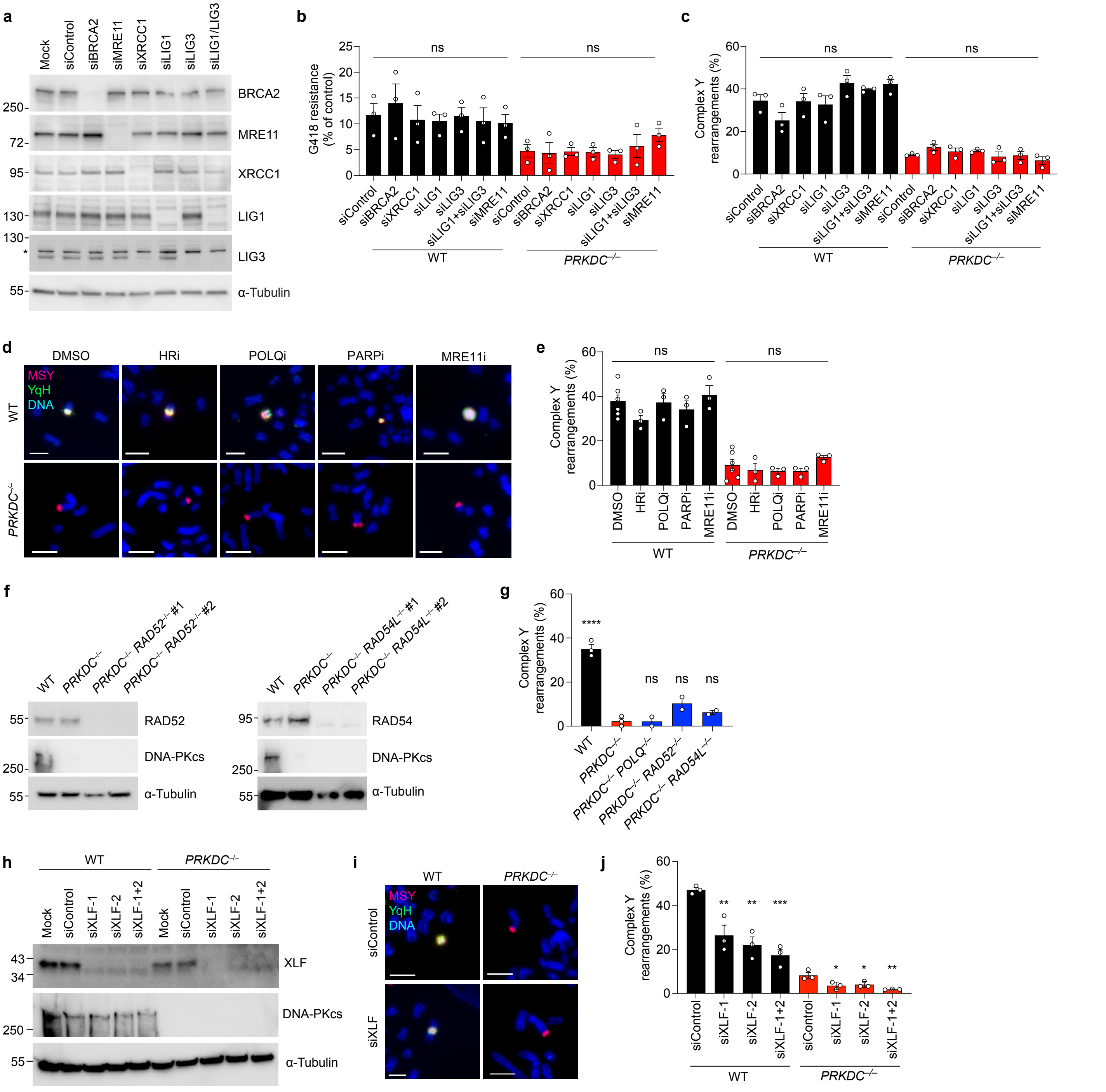

Supplementary Fig. 6. Complex rearrangements in DNA-PKcs-deficient cells arise from functional redundancies of the NHEJ pathway.
a) Immunoblot confirmation of protein depletion using the indicated siRNAs. Asterisk represents non-specific band. **b)** Viability of WT and DNA-PKcs KO cells (clone #1) upon siRNA-mediated depletion of the indicated DSB repair factors, treatment with DOX/IAA, and selection with G418. Data represent mean ± SEM from n = 3 independent experiments. **c)** Frequency of complex rearrangements in WT cells and DNA-PKcs KO cells upon siRNA-mediated depletion of indicated DSB repair factors, treatment with DOX/IAA, and selection with G418. Data represent mean ± SEM from n = 3 independent experiments pooled from (left to right): 90, 80, 98, 105, 95, 88, 87, 100, 74, 102, 101, 109, 114, and 109 metaphase spreads with Y chromosome rearrangements. **d)** Representative metaphase FISH images hybridized to the indicated probes from WT and DNA-PKcs KO cells with Y chromosome rearrangements after different small inhibitor treatment. Scale bar, 5 μm. **e)** Frequency of complex rearrangements in WT and DNA-PKcs KO cells after treatment with the indicated small molecule inhibitors. Data represent mean ± SEM from n = 6 (DMSO) or n = 3 (inhibitor treatment) independent experiments pooled from (left to right): 261, 154, 158, 156, 108, 256, 160, 157, 159 and 71 metaphase spreads with Y chromosome rearrangements. **f)** Immunoblot confirmation of double KO clones. **g)** Frequency of complex rearrangements in double KO cells. Data represent mean ± SEM from n = 3 independent experiments for WT and DNA-PKcs KO cells and n = 2 clones per double KO genotype pooled from (left to right): 177, 147, 128, 135, and 112 metaphase spreads containing Y chromosome rearrangements. **h)** Immunoblot confirmation of XLF depletion using the indicated siRNAs. **i)** Representative metaphase FISH images hybridized to the indicated probes from WT and DNA-PKcs KO cells with Y chromosome rearrangements after depletion of XLF. Scale bar, 5 μm. **j)** Frequency of complex rearrangements in WT and DNA-PKcs KO cells after depletion of XLF. Data represent mean ± SEM from n = 3 independent experiments pooled from (left to right): 134, 106, 95, 132, 152, 173, 180, and 168 metaphase spreads containing Y chromosome rearrangements. Statistical analyses for all plots were calculated by ordinary one-way ANOVA test with multiple comparisons. ns, not significant; *$p \leq 0.05$; **$p \leq 0.01$; ***$p \leq 0.001$; ****$p \leq 0.0001$. Molecular weight markers in panels **(a)**, **(f)**, and **(h)** are indicated in kilodaltons. Source data are provided as a Source Data file.

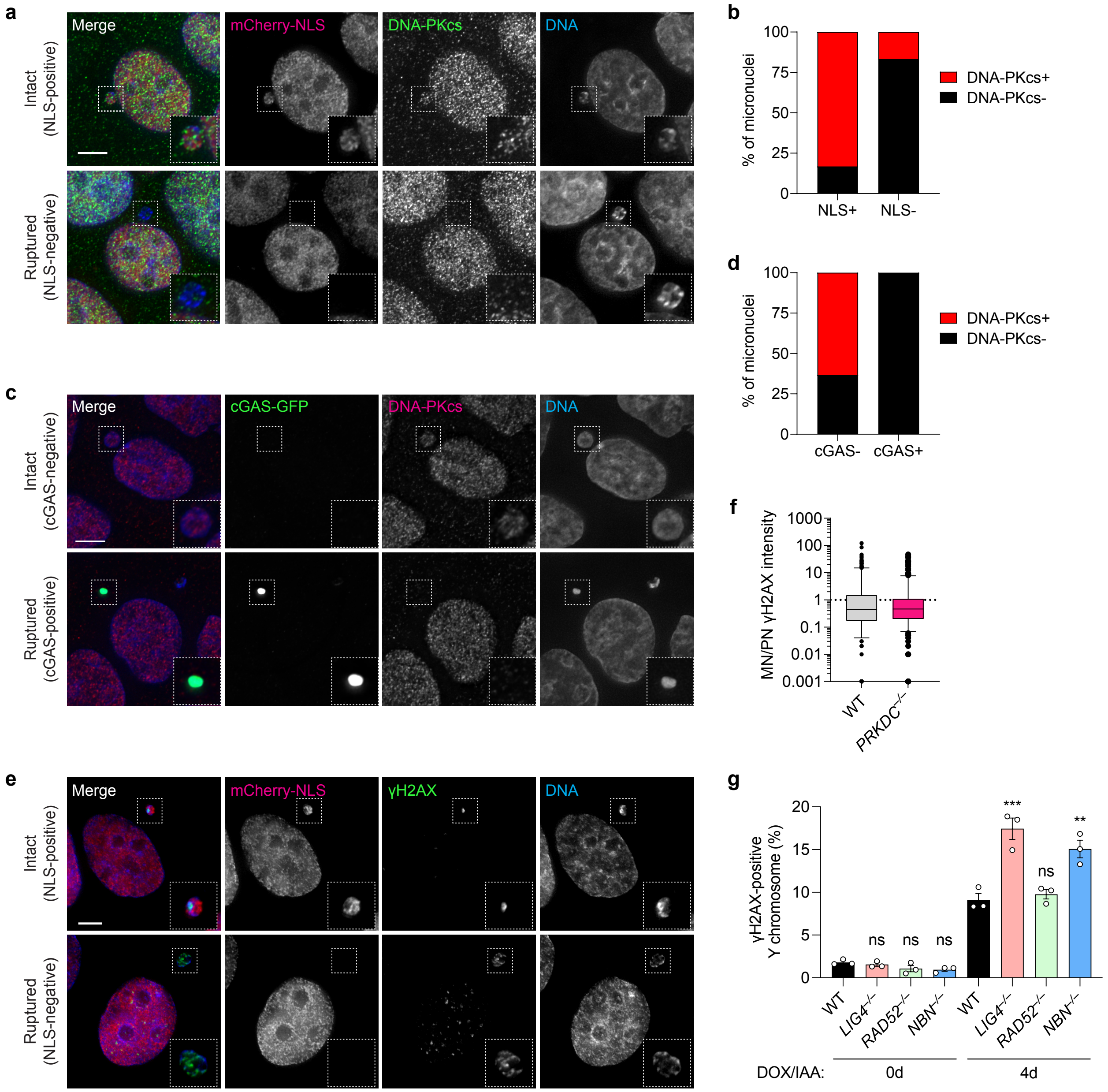

Supplementary Fig. 7. Nuclear envelope rupture triggers the loss of DNA-PKcs from micronuclei.
a) Representative immunofluorescent images of DNA-PKcs in DLD-1 cells expressing mCherry fused to a nuclear localization signal (NLS) with intact (NLS-positive) or ruptured (NLS-negative) micronuclei. **b)** Proportion of intact and ruptured micronuclei with detectable levels of DNA-PKcs. Data represent n = 149 interphase cells with micronuclei. **c)** Representative immunofluorescent images of DNA-PKcs in DLD-1 cells expressing cGAS-GFP with intact (cGAS-negative) or ruptured (cGAS-positive) micronuclei. **d)** Proportion of intact and ruptured micronuclei with detectable levels of DNA-PKcs. Data represent n = 151 interphase cells with micronuclei. **e)** Representative immunofluorescent images of γH2AX in DNA-PKcs KO DLD-1 cells expressing mCherry-NLS with intact or ruptured micronuclei. **f)** Fluorescence intensity of γH2AX in WT and DNA-PKcs KO micronuclei compared to their corresponding primary nucleus. Data represent 10-90 percentile from n = 207 interphase cells with micronuclei for both WT and DNA-PKcs KO. Scale bar for panels **(a)**, **(c)**, and **(e)**, 5 µm. **g)** Frequency of Y chromosomes marked by extensive γH2AX in the nucleus before and after 4-day DOX/IAA treatment. Data represent the mean ± SEM of n = 3 independent experiments from (left to right): 615, 657, 695, 699, 570, 523, 691, and 562 cells. Statistical analyses were calculated by ordinary one-way ANOVA test with multiple comparisons. ns, not significant; **$p \le 0.01$; ***$p \le 0.001$. Source data are provided as a Source Data file.

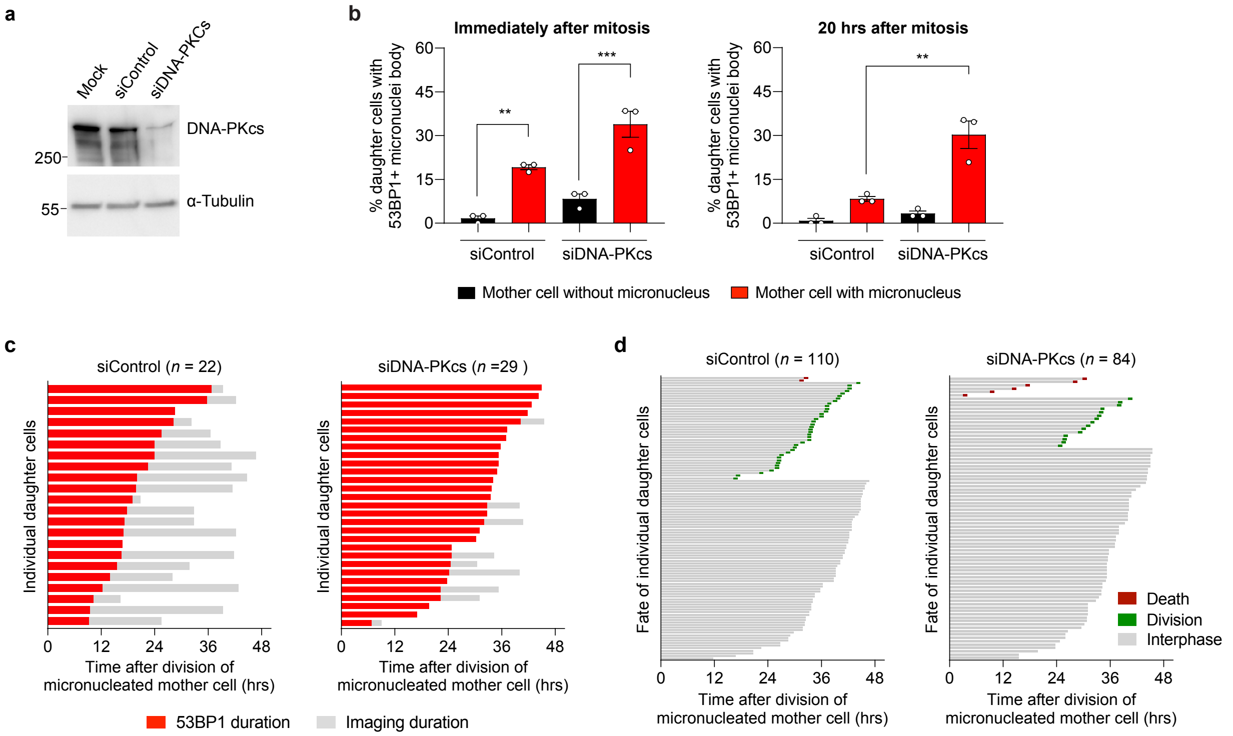

Supplementary Fig. 8. Depletion of DNA-PKcs prolongs 53BP1 residence time at MN bodies and triggers cell cycle arrest.
a) Immunoblot confirmation of DNA-PKcs depletion by siRNAs. **b)** Frequency of Halo-53BP1-labeled MN body formation and persistence. Data represent mean ± SEM of *n* = 3 independent experiments pooled from (left to right): 60, 55, 60, and 42 micronucleated mother cells. Statistical analyses were calculated by ordinary one-way ANOVA test with multiple comparisons. ns, not significant; ***p* ≤ 0.01; ****p* ≤ 0.001. **c)** Duration of Halo-53BP1 residence time from panel **(b)** after MN body formation in individual daughter cells with or without siRNA-mediated depletion of DNA-PKcs. **d)** Fate of daughter cells after division of micronucleated mother cells upon siRNA-mediated depletion of DNA-PKcs. Sample sizes in panels **(c-d)** represent individual daughter cells. Source data are provided as a Source Data file.

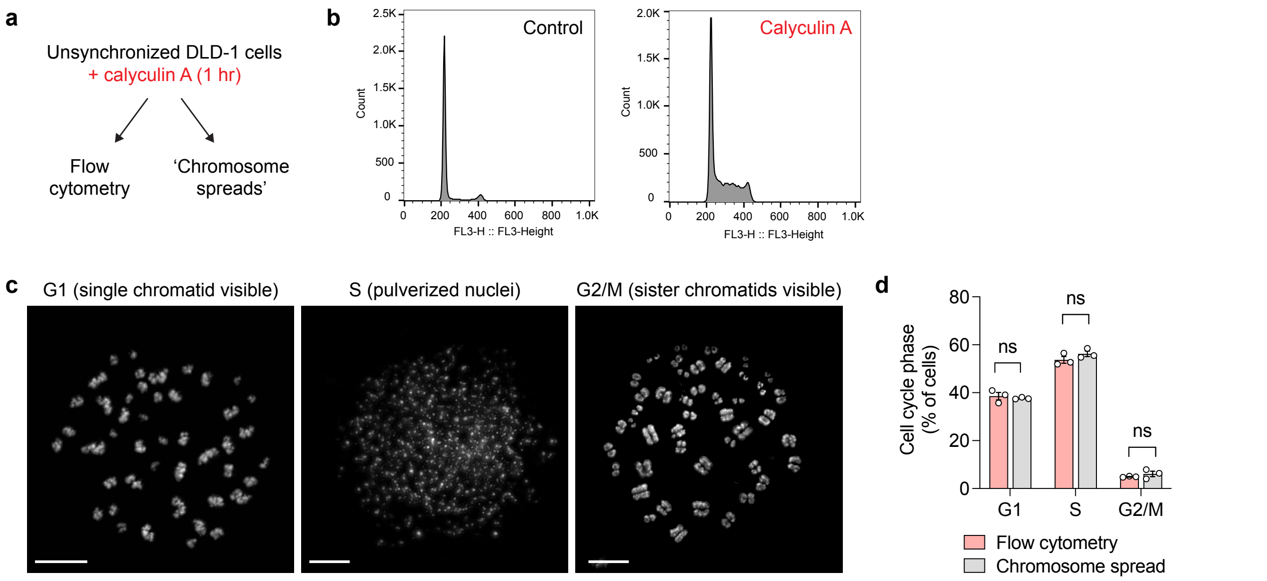

Supplementary Fig. 9. Characterization of calyculin A-induced premature chromosome condensation.
a) Unsynchronized DLD-1 cells were stimulated with calyculin A and analyzed for cell cycle stage by flow cytometry or preparation of chromosome spreads. **b)** Representative cell cycle profiles by flow cytometry. **c)** Examples of 'metaphase-like' chromosome spreads following induction of premature chromosome condensation with calyculin A for cells in the indicated cell cycle stages. Scale bar, 10 µm. **d)** Comparison of cell cycle distribution by flow cytometry versus microscopic analysis of chromosome spreads. Data represent mean ± SEM of n = 3 independent experiments from 53,326 flow-sorted cells and 1,103 chromosome spreads. Statistical analyses were calculated by two-tailed unpaired Student's t-test as indicated. ns, not significant. Source data are provided as a Source Data file.

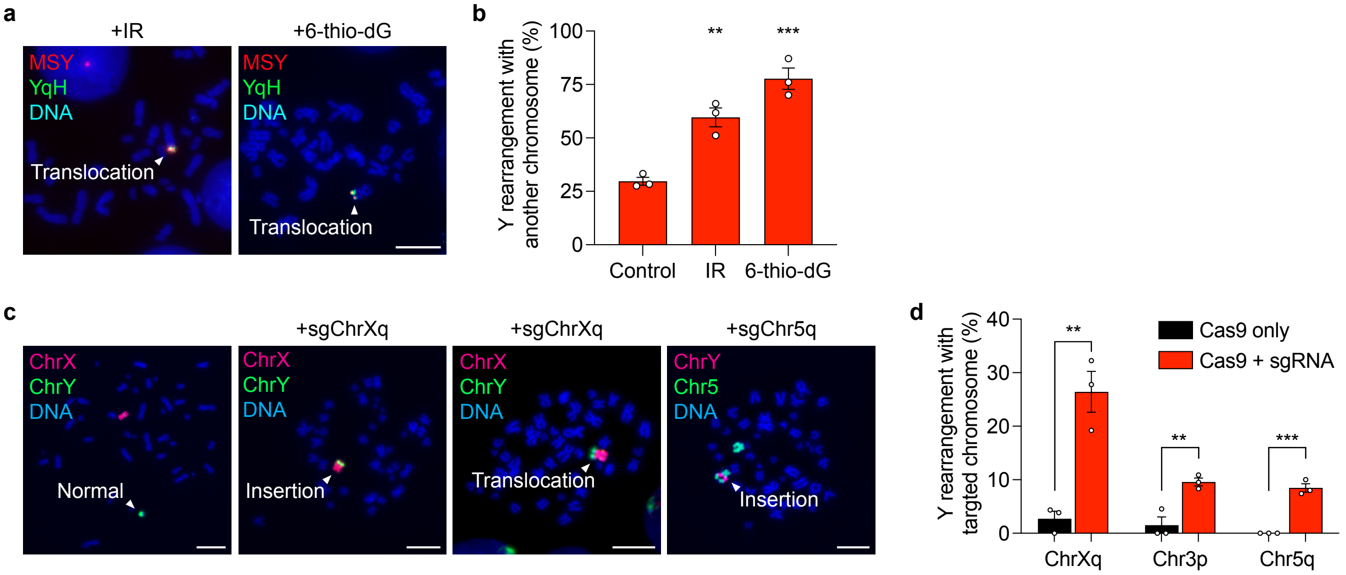

Supplementary Fig. 10. Induction of genome-wide or Cas9-induced DNA breaks on nuclear chromosomes facilitates translocations involving the micronucleated chromosome.

a) Representative images of metaphase spreads hybridized to the indicated FISH probes showing examples of Y chromosome translocations. Scale bar, 10 µm. **b)** Frequency of inter-chromosomal rearrangements in cells following 3d DOX/IAA treatment, exposure to IR (2 Gy) or 6-thio-dG, and G418 selection. Data represent mean ± SEM of *n* = 3 independent experiments from (left to right): 222, 231, and 152 metaphase spreads with Y chromosome rearrangements. **c)** Representative images of metaphase spreads hybridized to the indicated FISH probes showing examples of normal or rearranged Y chromosomes with the indicated translocation partner. Scale bar, 10 µm. **d)** Frequency of Y chromosome rearrangements with the CRISPR/Cas9-targeted chromosome. Data represent mean ± SEM of *n* = 3 independent experiments from (left to right): 60, 75, 78,115, 84, and 77 metaphase spreads with Y chromosome rearrangements. Statistical analyses were calculated by ordinary one-way ANOVA test with multiple comparisons for panel **(b)** and by two-tailed unpaired Student's t-test for panel **(d)**. $^{**}p \leq 0.01$; $^{***}p \leq 0.001$. Source data are provided as a Source Data file.

Supplementary Table 1. List of oligonucleotides sequences used in this study.

Gene	Sequence (5' to 3') or Source
NHEJ1 – sgRNA 1	ctctttaggagctgaacaag
NHEJ1 – sgRNA 2	ggagaaggtagcttcgcta
PRKDC – sgRNA 1	tccccttctggcatctgcg
PRKDC – sgRNA 2	gaaacgtccgcttatagagc
LIG4 – sgRNA 1	agaacacccactggaactca
LIG4 – sgRNA 2	ttgcttaatttacctagaga
LIG4 – sgRNA 3	gaaagagagagaatggccta
TP53BP1 – sgRNA 1	cagaatcatcctctagaacc
TP53BP1 – sgRNA 2	acgaggagacggtaatagt
POLQ – sgRNA 1	gtagagttcagcattcaacc
POLQ – sgRNA 2	ctgactccaaaagcggtaca
RAD52 – sgRNA 1	gtgctacattgagggtcatc
RAD52 – sgRNA 2	tccatcacgcagcagaatgt
RAD54L – sgRNA 1	gtcaccagtcggcgcatccc
RAD54L – sgRNA 2	gtgcaagccagaaattgaca
NBN – sgRNA 1	caagaagagcatgcaaccaa
NBN – sgRNA 2	agaatgcactcaccttgtca
NHEJ1 – primer 1	ccttcgtgttaaccagggct
NHEJ1 – primer 2	accatccagggtctacctca
PRKDC – primer 1	taaacattgctgacctcctggt
PRKDC – primer 2	tcttccctttgtgaaagactacg
LIG4 – primer 1	tcagacacttcagggaatttttaga
LIG4 – primer 2	tgctcaagtgctgaactctga
TP53BP1 – primer 1	cattccaggggagcagatgg
TP53BP1 – primer 2	cgcagataccacagtaggctt
POLQ – primer 1	gttggcatgagtgctaccct
POLQ – primer 2	cttcacttgtagcatgggc
RAD52 – primer 1	gtgtggtaaggaattaacacagctt
RAD52 – primer 2	gcagcaggtctactccatcc
RAD54L – primer 1	aacgaggtatgggctatggg
RAD54L – primer 2	gccatctttgttagggctcc
NBN – primer 1	gccatctctgcaactctgatac
NBN – primer 2	tgtcataaccttctcggtgga
Control – siRNA	CUACAUCCCGAUCGAUGAUdTdT
XLF – siRNA1	GCAUUACAGUGCCAAGUGAdTdT
XLF – siRNA2	CGCUGAUUCGAGAUCGAUUGAdTdT
siControl	Dharmacon ON-TARGETplus siRNA (D-001810-10-05)
siMRE11	Dharmacon ON-TARGETplus siRNA (L-009271-00-0005)
siBRCA2	Dharmacon ON-TARGETplus siRNA (L-003462-00-00050)

siXRCC1	Dharmacon ON-TARGETplus siRNA (L-009394-00-0005)
siLIG1	Dharmacon ON-TARGETplus siRNA (L-011076-00-0005)
siLIG3	Dharmacon ON-TARGETplus siRNA (L-009227-00-0005)
siDNA-PKcs	Dharmacon ON-TARGETplus siRNA (L-005030-00-0005)

Supplementary Table 2. List of primary antibodies used in this study.

Antibody	Source
α-Tubulin	Cell Signaling 3873
53BP1	Novus NB100-304
BRCA2	Cell Signaling 10741
LIG4	Cell Signaling 14649
DNA-PKcs	Abcam ab44815 (a gift from Benjamin Chen)
FLAG	Sigma F1804
GFP	Cell Signaling 2555
Histone H2A.X (phospho S139)	Cell Signaling 2577
Histone H2A.X (phospho S139)	Millipore 05-636
LIG1	Proteintech 18051-1-AP
LIG3	Fisher Scientific A301637AT
mCherry	Cell Signaling 43590S
MRE11	Proteintech 10744-1-AP
NBS1	Cell Signaling 14956
RAD52	Santa Cruz sc-365341
RAD54	Cell Signaling 15016
XRCC1	Proteintech 21468-1-AP
XLF	Cell Signaling 2854

Supplementary Figure 1c (also see next page)

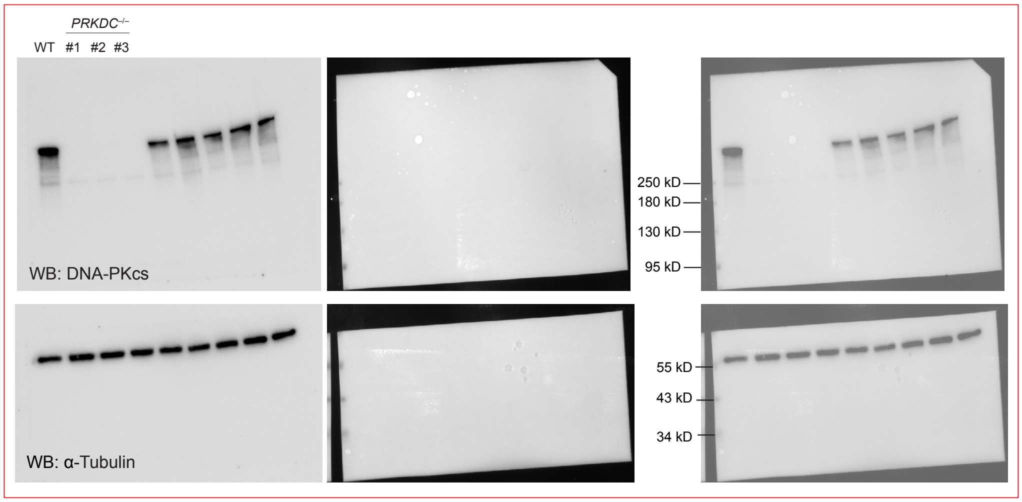

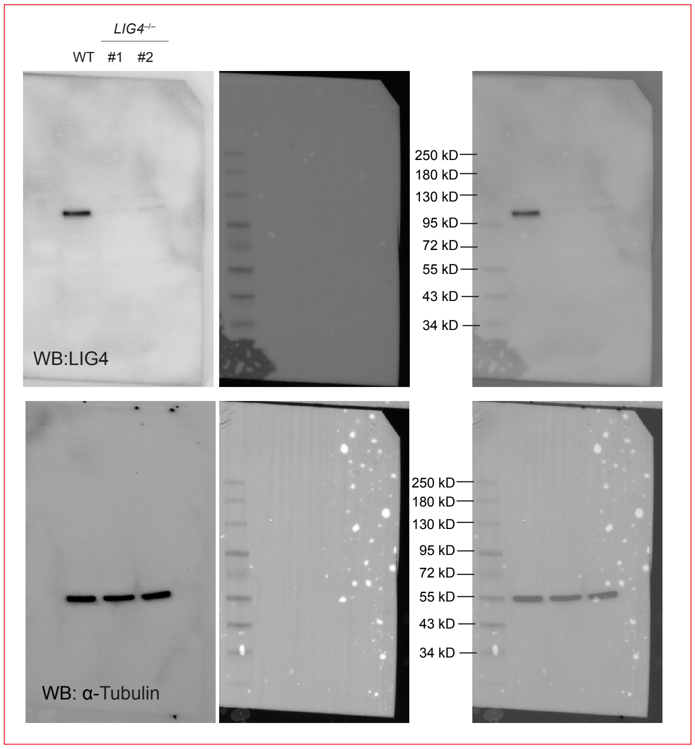

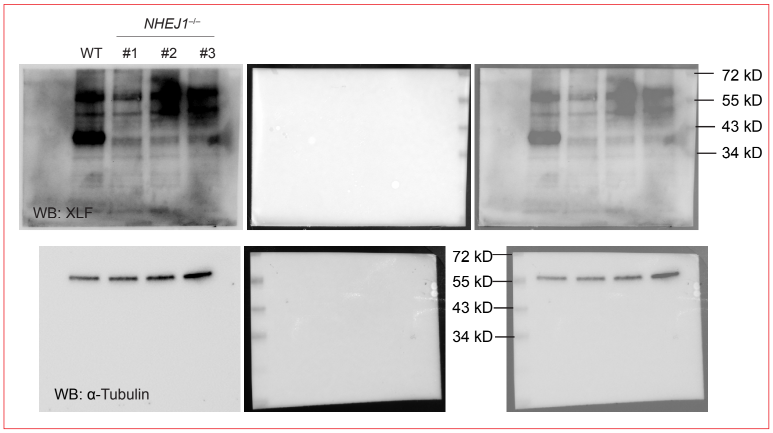

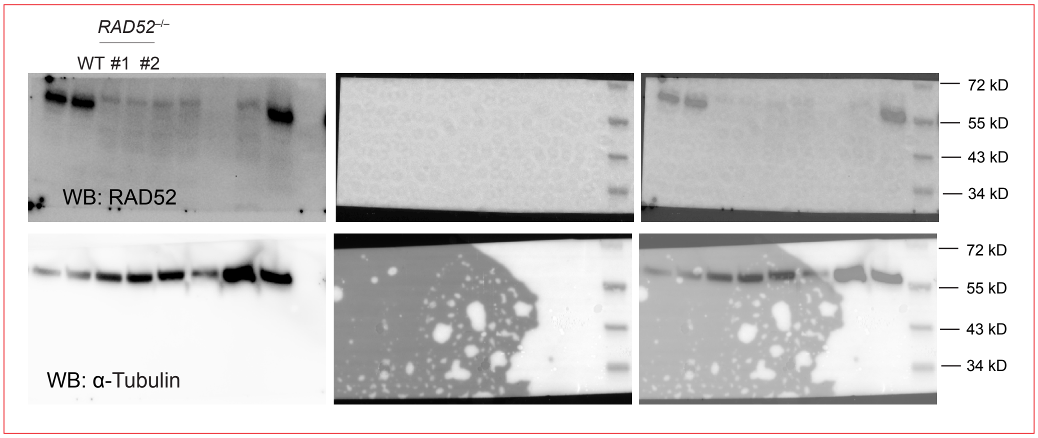

Supplementary Figure 1c (continued)

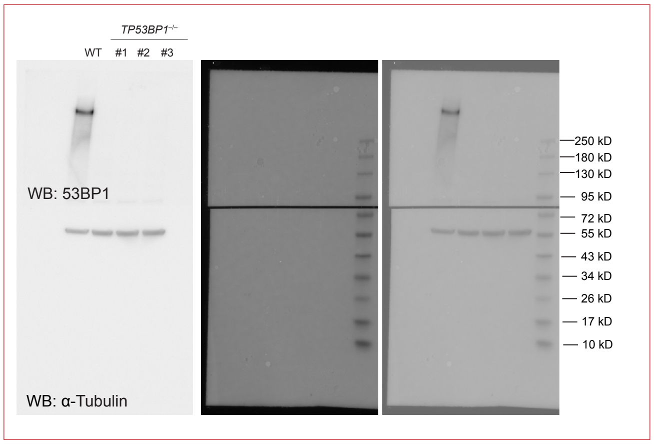

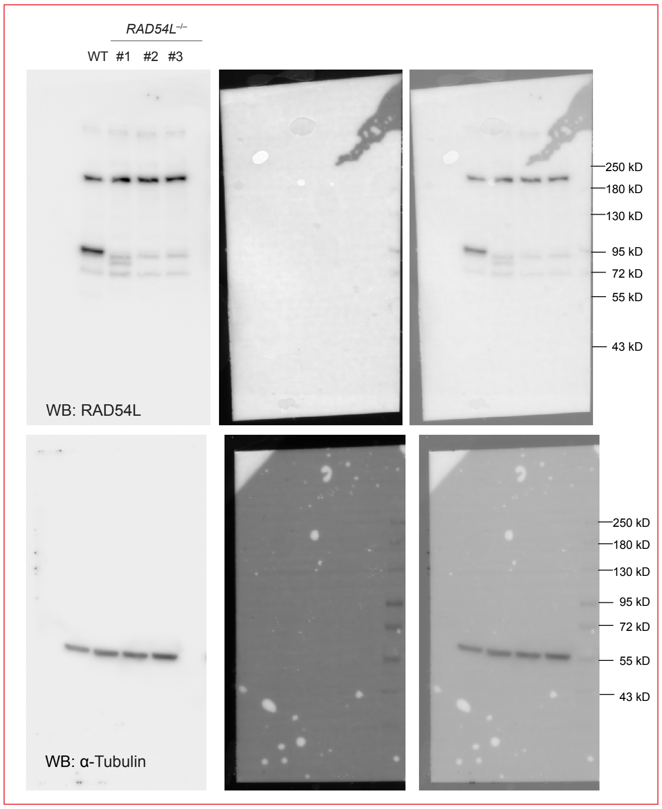

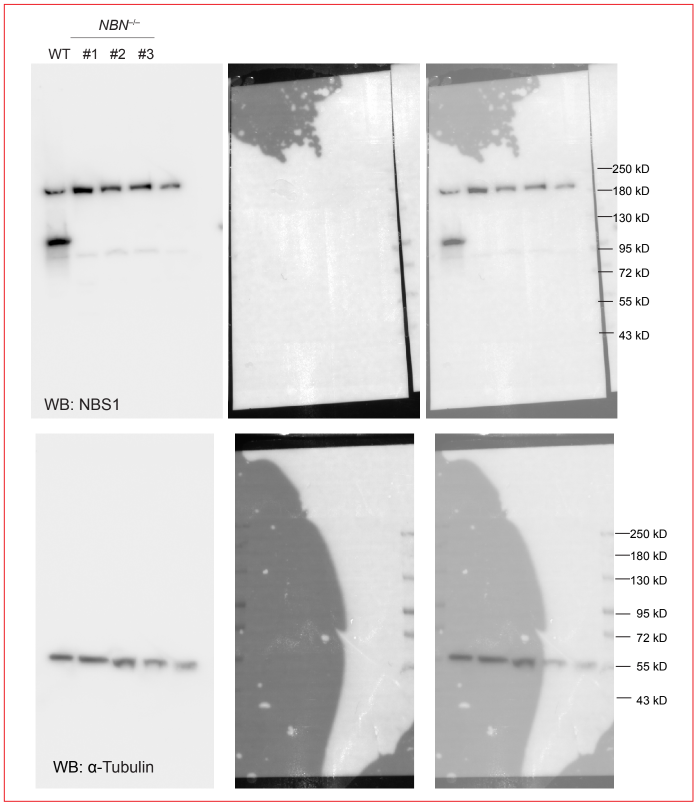

Supplementary Figure 5a

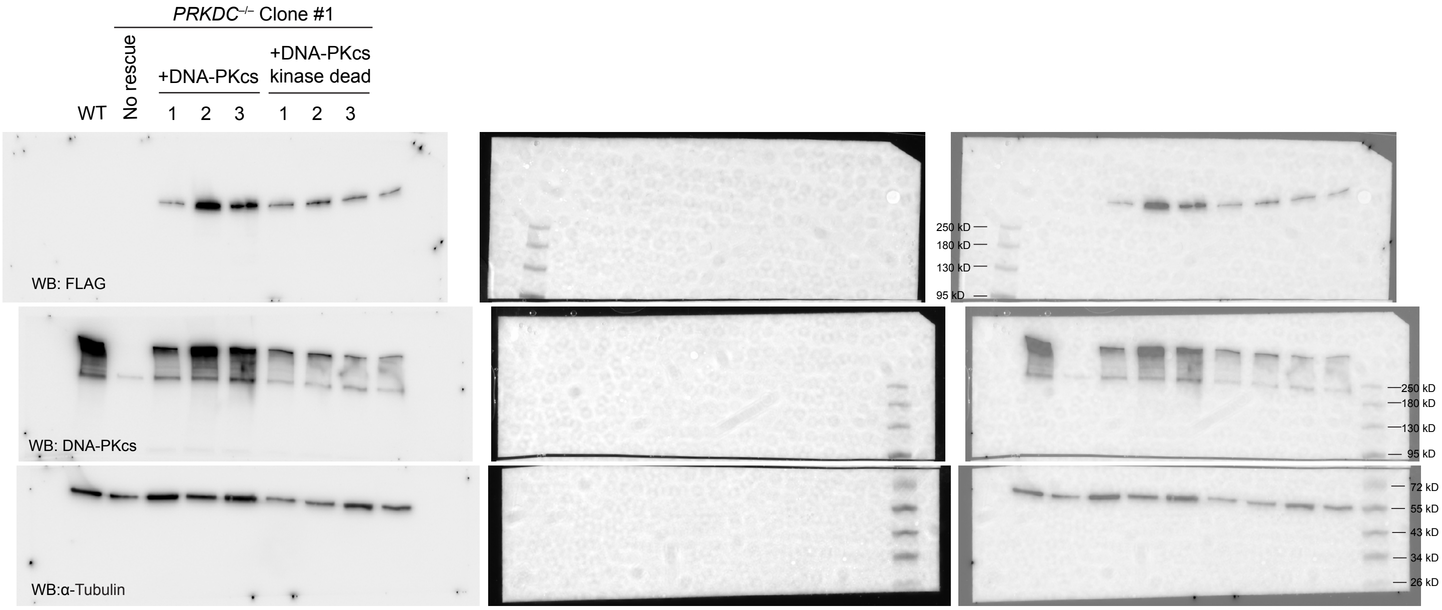

Supplementary Figure 6a

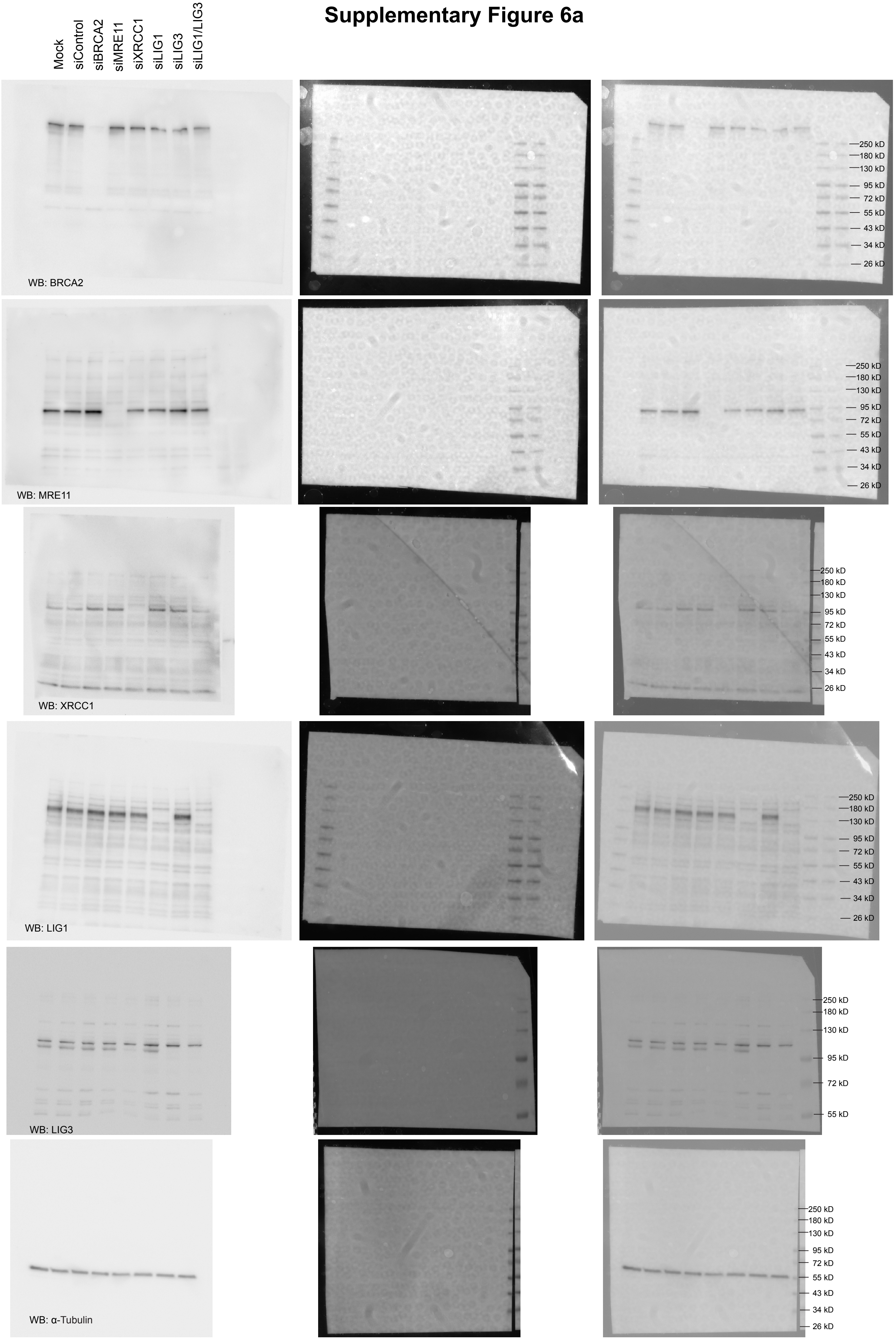

Supplementary Figure 6f

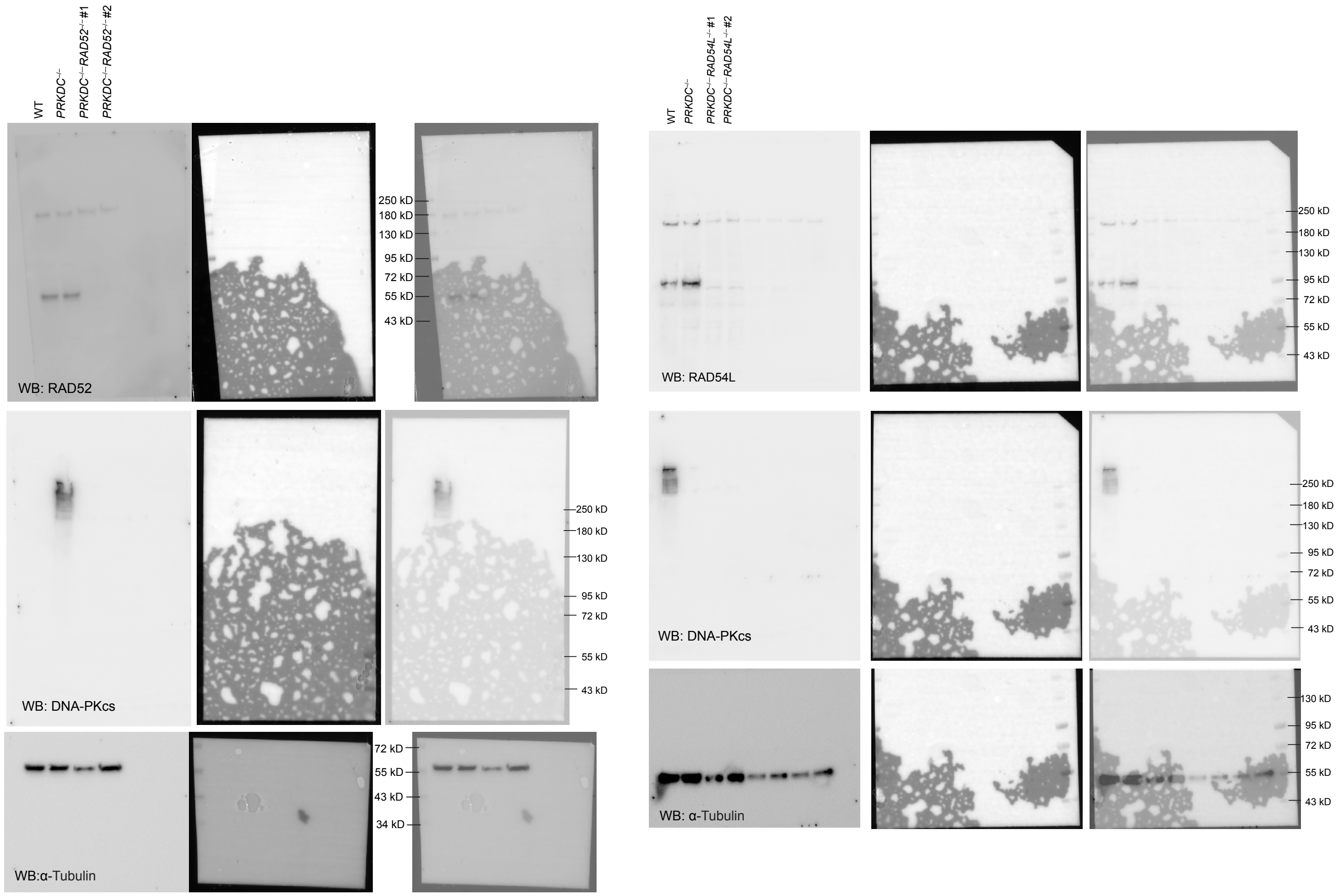

Supplementary Figure 6h

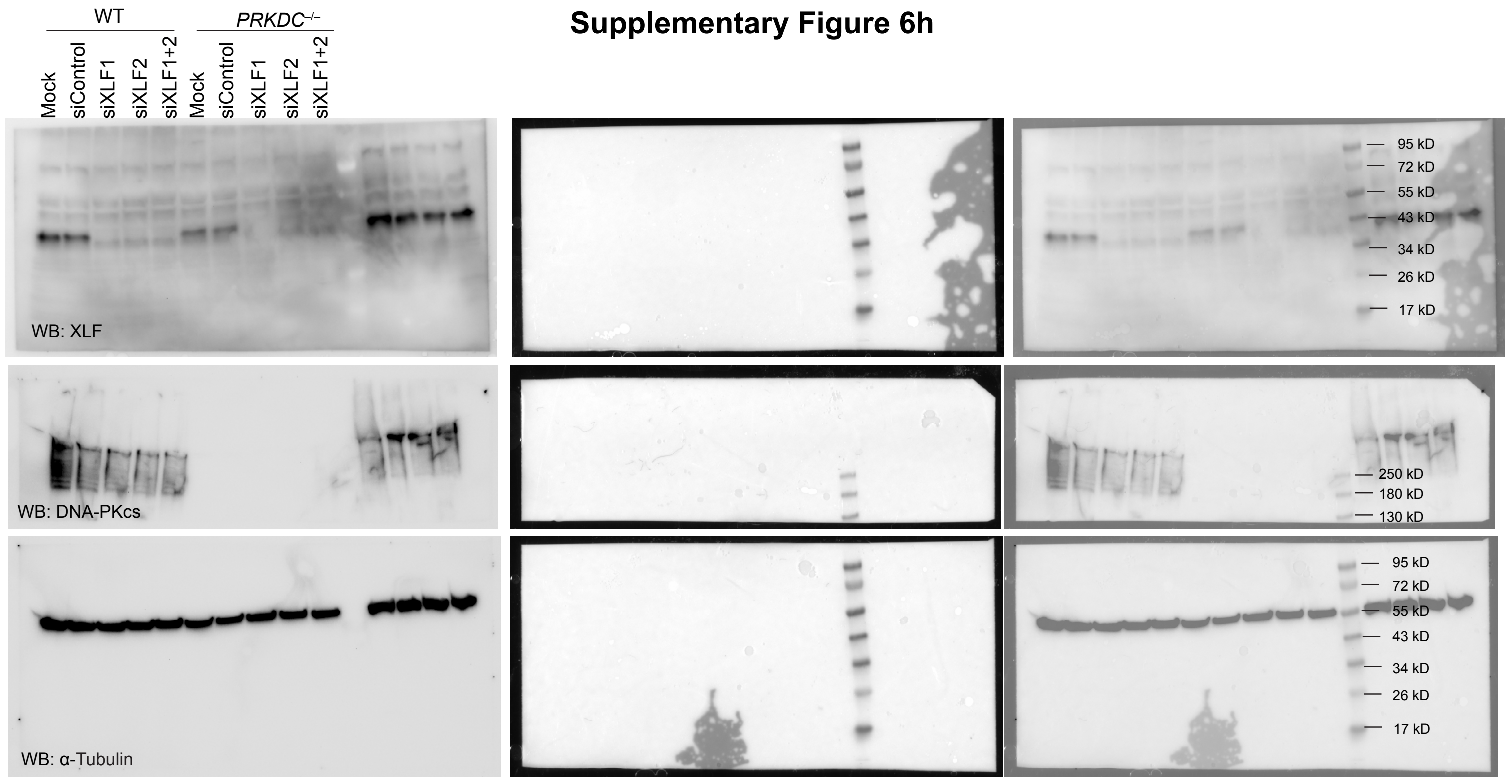

Supplementary Figure 8a

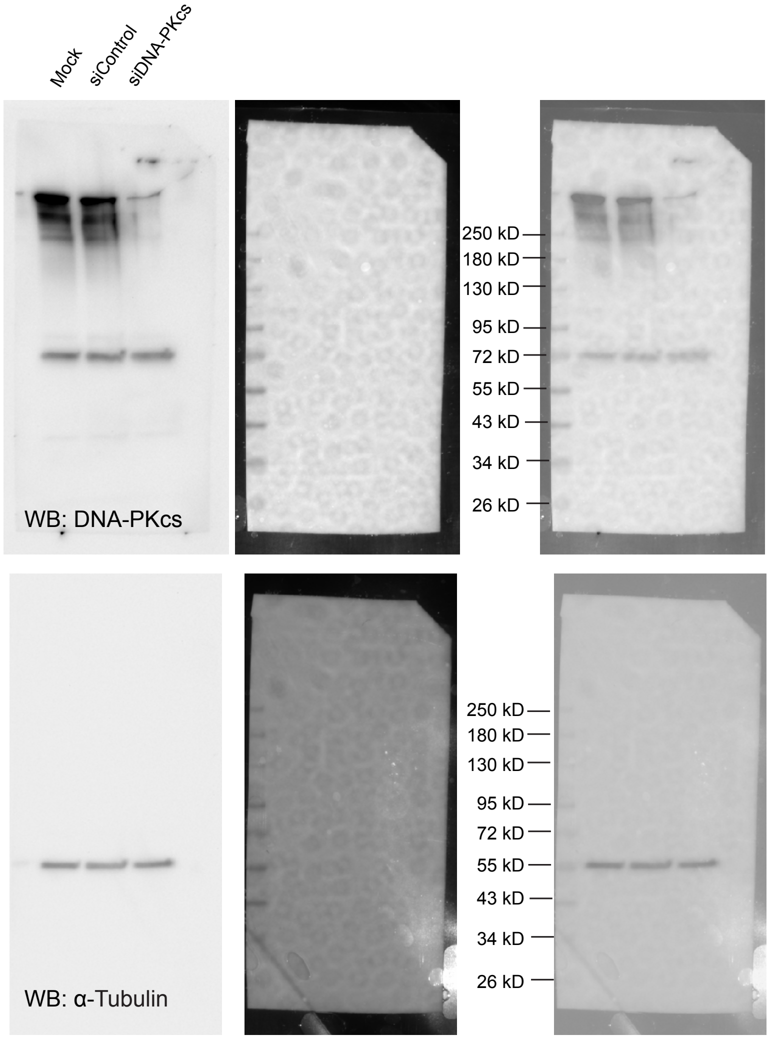

FACS Gating Strategies

Supplementary Fig. 1f – DR-GFP assay

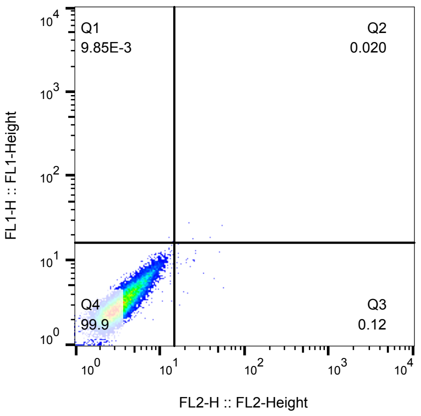

Negative control

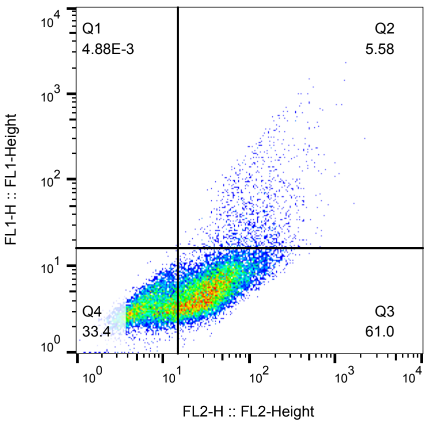

Example plot from one sample after transfection

Q1 (GFP positive)
Q2 (GFP/RFP double positive)
Q3 (RFP positive)
Q4 (Negative)

Supplementary Fig. 9b – Cell cycle profiling

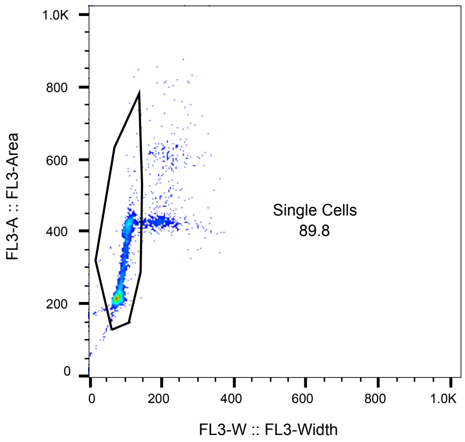

Doublet exclusion

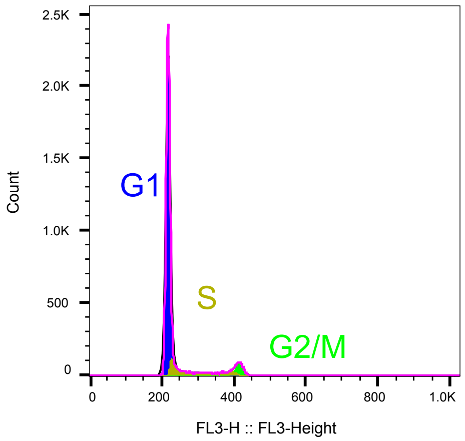

Cell cycle fitting
