# Supplementary material for: Non-homologous end joining shapes the genomic rearrangement landscape of chromothripsis from mitotic errors
Source: Nat Commun. 2024 Jul 4;15:5611. doi: 10.1038/s41467-024-49985-5 (PMC11224358; doi:10.1038/s41467-024-49985-5)
Supplement: Supplementary file 3 — Reporting Summary [file 41467_2024_49985_MOESM3_ESM.pdf]

nature portfolio

Corresponding author(s): Peter Ly

Last updated by author(s): Apr 23, 2024

Reporting Summary

Nature Portfolio wishes to improve the reproducibility of the work that we publish. This form provides structure for consistency and transparency in reporting. For further information on Nature Portfolio policies, see our Editorial Policies and the Editorial Policy Checklist.

Statistics

For all statistical analyses, confirm that the following items are present in the figure legend, table legend, main text, or Methods section.

n/a	Confirmed	
☐	☒	The exact sample size (*n*) for each experimental group/condition, given as a discrete number and unit of measurement
☐	☒	A statement on whether measurements were taken from distinct samples or whether the same sample was measured repeatedly
☐	☒	The statistical test(s) used AND whether they are one- or two-sided<br>*Only common tests should be described solely by name; describe more complex techniques in the Methods section.*
☒	☐	A description of all covariates tested
☐	☒	A description of any assumptions or corrections, such as tests of normality and adjustment for multiple comparisons
☐	☒	A full description of the statistical parameters including central tendency (e.g. means) or other basic estimates (e.g. regression coefficient) AND variation (e.g. standard deviation) or associated estimates of uncertainty (e.g. confidence intervals)
☐	☒	For null hypothesis testing, the test statistic (e.g. *F*, *t*, *r*) with confidence intervals, effect sizes, degrees of freedom and *P* value noted<br>*Give P values as exact values whenever suitable.*
☒	☐	For Bayesian analysis, information on the choice of priors and Markov chain Monte Carlo settings
☒	☐	For hierarchical and complex designs, identification of the appropriate level for tests and full reporting of outcomes
☒	☐	Estimates of effect sizes (e.g. Cohen's *d*, Pearson's *r*), indicating how they were calculated

Our web collection on statistics for biologists contains articles on many of the points above.

Software and code

Policy information about availability of computer code

Data collection	Microscopy images were collected on a DeltaVision Ultra microscope system (GE Healthcare), Metafer Scanning and Imaging Platform (MetaSystems, Metafer 4), or ImageXpress Confocal HT.ai High-Content Imaging System (Molecular Devices), as described in the Methods. Whole-genome sequencing was performed by Novogene on an Illumina NovaSeq. Flow cytometry samples were examined using FACSCalibur flow cytometer (BD Biosciences).
Data analysis	Statistical analyses for cell biological experiments were performed with GraphPad Prism (version 10.1.1) using the tests described in the figure legends. Microscopy data were analyzed with Fiji (v.2.1.0/1.53c), softWoRx (v.7.2.1, Cytiva), and Isis Fluorescence Imaging Platform (v3.13.6, MetaSystems). Whole-genome sequencing data were analyzed as described in the Methods with the following tools: SAMtools (v1.12), Sambamba (v0.8.1), Mosdepth (v0.3.1), Control-FREEC (v11.6), SAGE (v2.8.0), and MutationalPatterns Bioconductor package (v3.10). Flow cytometry data were analyzed with FlowJo (v.10.8.2, BD Biosciences).

For manuscripts utilizing custom algorithms or software that are central to the research but not yet described in published literature, software must be made available to editors and reviewers. We strongly encourage code deposition in a community repository (e.g. GitHub). See the Nature Portfolio guidelines for submitting code & software for further information.

Data

Policy information about availability of data

All manuscripts must include a data availability statement. This statement should provide the following information, where applicable:

- Accession codes, unique identifiers, or web links for publicly available datasets
- A description of any restrictions on data availability
- For clinical datasets or third party data, please ensure that the statement adheres to our policy

Whole-genome sequencing data presented in this manuscript have been deposited at the European Nucleotide Archive under project accession ID PRJEB64431.

Research involving human participants, their data, or biological material

Policy information about studies with human participants or human data. See also policy information about sex, gender (identity/presentation), and sexual orientation and race, ethnicity and racism.

Reporting on sex and gender	N/A
Reporting on race, ethnicity, or other socially relevant groupings	N/A
Population characteristics	N/A
Recruitment	N/A
Ethics oversight	N/A

Note that full information on the approval of the study protocol must also be provided in the manuscript.

Field-specific reporting

Please select the one below that is the best fit for your research. If you are not sure, read the appropriate sections before making your selection.

☒ Life sciences ☐ Behavioural & social sciences ☐ Ecological, evolutionary & environmental sciences

For a reference copy of the document with all sections, see nature.com/documents/nr-reporting-summary-flat.pdf

Life sciences study design

All studies must disclose on these points even when the disclosure is negative.

Sample size	Sample sizes were determined based on current practices in the field. Exact sample sizes and/or the number of independent experiments performed per experiment are indicated in the figure, figure legends, or methods.
Data exclusions	No data were excluded from analyses.
Replication	All experiments were independently conducted multiple times or with multiple independent samples and clones as described in the figure legends.
Randomization	Data were not randomized during analyses.
Blinding	Investigators were not blinded during data collection and/or analysis as each series of experiments were performed by an individual researcher.

Reporting for specific materials, systems and methods

We require information from authors about some types of materials, experimental systems and methods used in many studies. Here, indicate whether each material, system or method listed is relevant to your study. If you are not sure if a list item applies to your research, read the appropriate section before selecting a response.

Materials & experimental systems

n/a	Involved in the study
☐	☒ Antibodies
☐	☒ Eukaryotic cell lines
☒	☐ Palaeontology and archaeology
☒	☐ Animals and other organisms
☒	☐ Clinical data
☒	☐ Dual use research of concern
☒	☐ Plants

Methods

n/a	Involved in the study
☒	☐ ChIP-seq
☐	☒ Flow cytometry
☒	☐ MRI-based neuroimaging

Antibodies

Antibodies used

Primary antibodies: XLF (Cell Signaling 2854), DNA-PKcs (Abcam ab44815), DNA Ligase IV (Cell Signaling 14649), 53BP1 (Novus NB100-304), RAD52 (Santa Cruz sc-365341), RAD54 (Cell Signaling 15016), NBS1 (Cell Signaling 14956), α-Tubulin (Cell Signaling 3873),Histone H2A.X (phospho S139) (Cell Signaling 2577), Histone H2A.X (phospho S139) (Millipore 05-636), FLAG (Sigma F1804), GFP (Cell Signaling 2555), mCherry (Cell Signaling 43590S), LIG1 (Proteintech 18051-1-AP), LIG3 (Fisher Scientific A301637AT), XRCC1 (Proteintech 21468-1-AP), MRE11 (Proteintech 10744-1-AP), and BRCA2 (Cell Signaling 10741). Secondary antibodies: Alexa Fluor-conjugated donkey anti-rabbit and donkey anti-mouse antibodies (Invitrogen) were used for immunofluorescence experiments, and horseradish peroxidase-conjugated goat anti-rabbit and donkey anti-mouse antibodies (Invitrogen) were used for immunoblotting.

Validation

All antibodies used in this study are commercially available. All critical antibodies were validated by immunoblotting of the knockout cells generated by CRISPR/Cas9 editing or siRNA knock down.

Manufacturer validation statements can be found at the links below.
XLF (Cell Signaling 2854, https://www.cellsignal.com/products/primary-antibodies/xlf-antibody/2854)
DNA-PKcs (Abcam ab44815, https://www.abcam.com/products/primary-antibodies/dna-pkcs-antibody-18-2-ab44815.html)
DNA Ligase IV (Cell Signaling 14649, https://www.cellsignal.com/products/primary-antibodies/dna-ligase-iv-d5n5n-rabbit-mab/14649)
53BP1 (Novus NB100-304, https://www.novusbio.com/products/53bp1-antibody_nb100-304)
RAD52 (Santa Cruz sc-365341, https://www.scbt.com/p/rad52-antibody-f-7)
RAD54 (Cell Signaling 15016, https://www.cellsignal.com/products/primary-antibodies/rad54-d4w3z-rabbit-mab/15016)
NBS1 (Cell Signaling 14956, https://www.cellsignal.com/products/primary-antibodies/p95-nbs1-d6j5i-rabbit-mab/14956)
α-Tubulin (Cell Signaling 3873, https://www.cellsignal.com/products/primary-antibodies/a-tubulin-dm1a-mouse-mab/3873)
Histone H2A.X (phospho S139) (Cell Signaling 2577, https://www.cellsignal.com/products/primary-antibodies/phospho-histone-h2a-x-ser139-antibody/2577)
Histone H2A.X (phospho S139) (Millipore 05-636, https://www.emdmillipore.com/US/en/product/Anti-phospho-Histone-H2A.X-Ser139-Antibody-clone-JBW301,MM_NF-05-636?)
FLAG (Sigma F1804, https://www.sigmaaldrich.com/US/en/product/sigma/f1804)
GFP (Cell Signaling 2555, https://www.cellsignal.com/products/primary-antibodies/gfp-antibody/2555)
mCherry (Cell Signaling 43590S, https://www.cellsignal.com/products/primary-antibodies/mcherry-e5d8f-rabbit-mab/43590)
LIG1 (Proteintech 18051-1-AP, https://www.ptglab.com/products/LIG1-Antibody-18051-1-AP.htm)
LIG3 (Fisher Scientific A301637AT, https://www.fishersci.com/shop/products/dnl3-polyclonal-antibody-bethyl-laboratories/501560878)
XRCC1 (Proteintech 21468-1-AP, https://www.ptglab.com/products/XRCC1-Antibody-21468-1-AP.htm)
MRE11 (Proteintech 10744-1-AP, https://www.ptglab.com/products/MRE11A-Antibody-10744-1-AP.htm)
BRCA2 (Cell Signaling 10741, https://www.cellsignal.com/products/primary-antibodies/brca2-d9s6v-rabbit-mab/10741)
Alexa Fluor-conjugated donkey anti-rabbit and donkey anti-mouse antibodies (Invitrogen, https://www.thermofisher.com/antibody/secondary/query/alexa%20fluor)
Horseradish peroxidase-conjugated goat anti-rabbit and donkey anti-mouse antibodies (Invitrogen, https://www.thermofisher.com/antibody/secondary/query/hrp)

Eukaryotic cell lines

Policy information about cell lines and Sex and Gender in Research

Cell line source(s)

DLD-1, human colorectal cancer cells; 293GP, human embryonic kidney cells.

Authentication

Cell lines were authenticated by morphological characteristics, SNP array analysis, karyotyping, and/or whole-genome DNA sequencing.

Mycoplasma contamination

Cell lines used in this study were routinely confirmed to be free of mycoplasma contamination using the Universal Mycoplasma Detection Kit (ATCC) and by routine DAPI staining.

Commonly misidentified lines
(See ICLAC register)

No commonly misidentified cell lines were used in this study.

Plants

Seed stocks	N/A
Novel plant genotypes	N/A
Authentication	N/A

Flow Cytometry

Plots

Confirm that:

- [x] The axis labels state the marker and fluorochrome used (e.g. CD4-FITC).
- [x] The axis scales are clearly visible. Include numbers along axes only for bottom left plot of group (a 'group' is an analysis of identical markers).
- [x] All plots are contour plots with outliers or pseudocolor plots.
- [x] A numerical value for number of cells or percentage (with statistics) is provided.

Methodology

Sample preparation	For cell cycle profiling assay, cells were collected fixed with 70% ethanol in PBS, and stained with propidium iodide as described in the methods. For HR reporter assay, cells were transfected with plasmids, collected by trypsinization, and resuspended in PBS with 10% FBS as indicated in the methods.
Instrument	Flow cytometry samples were examined using FACSCalibur flow cytometer (BD Biosciences).
Software	Flow cytometry data was analyzed with FlowJo (v.10.8.2, BD Biosciences).
Cell population abundance	For cell cycle profiling assay, cells were sorted to exclude doublets. The percentage of single cells after doublet exclusion was around 90%. For HR reporter assay, fluorescent positive cells were selected according to GFP/RFP channel, and the overall percentage of cells used for analysis was around 70%.
Gating strategy	For cell cycle profiling assay, doublets exclusion gating was conducted by plotting FSC width vs FCS area. The Watson model was used for cell cycle fitting. For HR reporter assay, non-transfected cells were used as a negative control, and GFP positive, RFP positive, or GFP/RFP double positive cells were separately gated and recorded for analysis.

- [x] Tick this box to confirm that a figure exemplifying the gating strategy is provided in the Supplementary Information.
